# Hemorrhagic pleural effusion secondary to sarcoidosis: A brief review

**DOI:** 10.4103/1817-1737.44783

**Published:** 2009

**Authors:** Santosh Kumar, Sanjay Kumar Verma, Rajni Singh, Rajendra Prasad

**Affiliations:** *Department of Pulmonary Medicine, C.S.M. Medical University, Lucknow, Uttar Pradesh, India*

**Keywords:** Hemorrhagic pleural effusion, sarcoidosis, non caseating granuloma

## Abstract

Pleural effusion is considered to be a rare manifestation of pulmonary sarcoidosis, but hemorrhagic effusion secondary to it is a very uncommon clinical presentation. This case is reported due to the rare manifestation in pulmonary sarcoidosis presenting clinically as hemorrhagic pleural effusion.

Sarcoidosis is a multisystem granulomatous disorder of unknown origin and commonly affects the intrathoracic lymph nodes in more than 90% of patients. The incidence of pleural effusion with sarcoidosis ranges from 0.7 to 10%. The pleural fluid is usually an exudate with a variable amount of cells, a major part being lymphocytes. The occurrence of hemorrhagic pleural effusion secondary to sarcoidosis is a very uncommon clinical presentation, as seen in the present case.

## Case Report

A 53-year-old female bidi smoker (pack year: 14) was admitted to our department with complaints of loss of appetite and left-sided chest pain for 5 months. On examination, the patient was alert and well oriented. She was not in obvious distress. Her vital signs were stable. The only significant finding on examination of the chest was reduced breath sound at the left lower axillary area with bi basilar crept in the infrascapular area.

**Figure 1:**
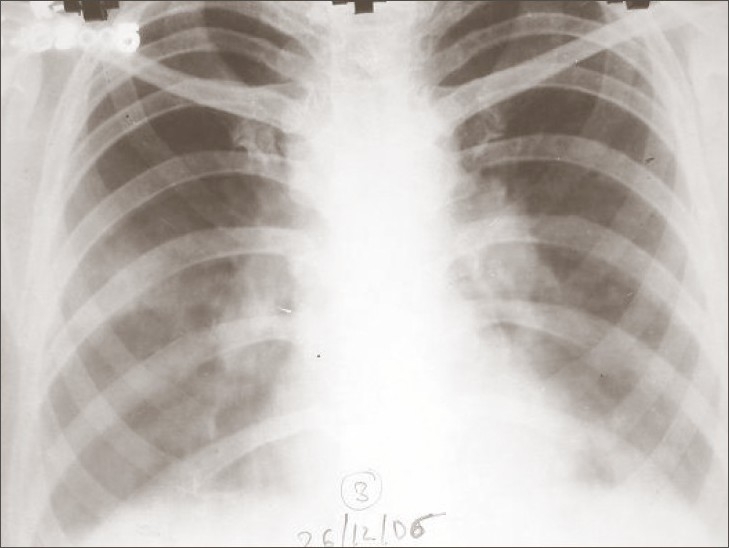
Chest X-ray revealed bilateral hilar prominence with left-sided pleural effusion

Her chest radiograph revealed bilateral hilar prominence with left-sided pleural effusion [Figure 1]. A left thoracentesis revealed thin, grossly hemorrhagic pleural fluid that did not clot and did not clear on sequential samples and had no obvious odor. Thus, considering the age of the patient (53 years) and her smoking habits and hemorrhagic pleural fluid, a malignant process was considered in the differential diagnosis.

**Figure 2:**
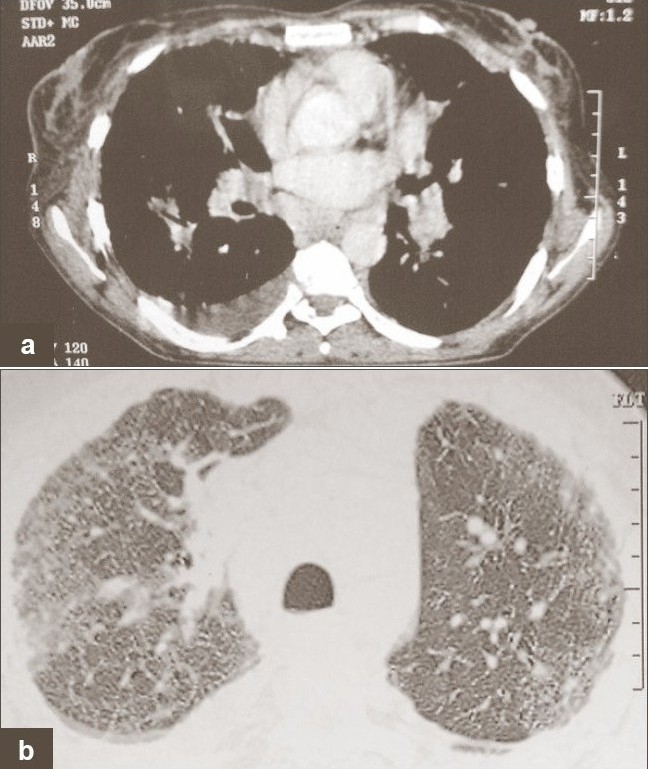
(a, b) Computed tomography revealed bilateral hilar lymphadenopathy with parenchymal opacity and left-sided pleural effusion

The pleural fluid was exudates with cytology showing 2,200 cells/cu mm with 88% lymphocytes and 12% neutrophils, red blood cell (RBC) count of 2,200,000/cu mm and pleural fluid hematocrit was 26%. Biochemical analysis revealed sugar 44 mg/dl, proteins 5.4 g/dl and adenosine deaminase 32 U/L (normal limit being less than 40 U/L). Pleural fluid cultures were negative for both pyogens and *M. tuberculosis*. Pleural fluid was negative for malignant cells on five consecutive occasions. Her pleural biopsy was performed by Abraham's needle, which was negative. Her PPD skin test was negative. A computerised tomographic scan of her chest revealed bilateral hilar lymphadenopathy with parenchymal opacity and left-sided pleural effusion [Figure 2a and Figure 2b].

Further, ultrasonography of the neck and abdomen, mammography and gynecological evaluation were performed to rule out any evidence of a malignant condition, but all were normal except for a deep cervical lymphadenopathy. An excisional biopsy of the upper deep cervical lymph node revealed a noncaseating granuloma. Serum ACE was 168 IU/L. Fiber optic bronchoscopy revealed no endobronchial abnormality. Bronchial brushing and bronchoalveolar lavage revealed predominance of lymphocytes and no malignant cells or AFB. A repeat thoracoscopic-guided pleural biopsy revealed a noncaseating granuloma.

Thus, a diagnosis of sarcoidosis presenting as hemorrhagic pleural effusion with bilateral hilar lymphadenopathy and deep cervical lymphadenopathy was made.

**Figure 3:**
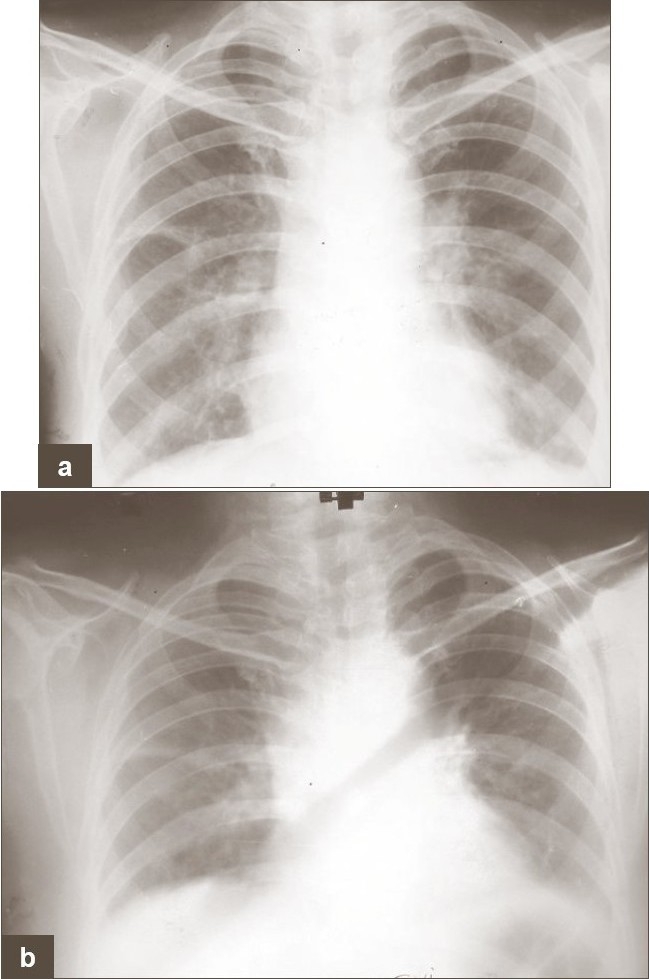
(a, b) Follow-up chest X-ray revealed resolution of pleural effusion as well as parenchymal shadows

She was put on prednisolone 40 mg/day followed by in-tapered dosages. Her appetitite was improved and chest pain subsided gradually and without recurrence of symptoms of pleurisy [Figure 3a, Figure 3b].

## Discussion

In sarcoidosis, the involvement of the pleura may present as pleural effusion, pneumothorax, pleural thickening, hydropneumothorax, trapped lung and chylothorax.[1–4] Pleural sarcoidosis as pleural effusion is still a rare manifestation of sarcoidosis in all published series and hemorrhagic effusion secondary to sarcoidosis is a very uncommon clinical presentation.[5] Apart from the present case, to the best of our knowledge, only three cases of hemorrhagic pleural effusion secondary to sarcoidosis were published in the literature.[5–7] The detailed features of all three cases and their comparison with the present case are shown in Table 1.

**Table 1:** Characters of three cases of hemorrhagic pleural effusion secondary to sarcoidosis were published in the literature

Investigations	Author			

	Paul De Vuyst (1979)	Takahashi N (1992)	Megumi (2002)	Present author
Number of cases	1	1	1	1
Age (years)	50	30	64	53
Sex	Male	Female	Male	Female
Smoking history	Nonsmoker	Data NA	Smoker	Smoker
Peripheral lymph node	No	No	No	Single upper deep cervical node present.
				^*^Biopsy revealed noncaseating granuloma
Serum ACE (IU/L)	Data NA	Data NA	22.7	140
Pleural effusion (right or left side)	B/L	Data NA	Right	Left
Pleural fluid characteristics				
Color	Bloody	Bloody	Bloody	Bloody
Pleural fluid (TLC)	Data NA	Data NA	Data NA	1,240
Pleural fluid (DLC)	L94, M5	Data NA	Data NA	L88, P12
Pleural fluid RBCs (/cu mm)	97,000	Data NA	Data NA	2,20,000
Pleural fluid hematocrit (%)	Data NA	Data NA	Data NA	26
Pleural fluid protein (gm/dL)	4.5	Data NA	6.7	5.4
Pleural fluid protein/ serum protein ratio	Data NA	Data NA	Data NA	0.6
Pleural fluid LDH (IU/L)	Data NA	Data NA	1,103	270
Pleural fluid LDH/ serum LDH ratio	Data NA	Data NA	Data NA	0.61
ADA	Data NA	Data NA	Not elevated	Not elevated
Malignant cells	Data NA	Data NA	Negative	Negative
Bronchoscopic biopsy findings	No lesions	Data NA	Data NA	No lesions
Pleural biopsy (by thoracoscopic or Abraham's needle biopsy)	Not performed	Data NA	Thoracoscopic-guided biopsy specimen revealed noncaseating granuloma	Thoracoscopic-guided biopsy biopsy specimen revealed noncaseating granuloma
Mediastiniscopic-guided lymph node biopsy	Done, which revealed noncaseating granuloma	Not done	Not done	Not done

The most common causes of hemorrhagic pleural effusion include tumor (both primary pleuro-parenchymal as well as metastasis), trauma (both iatrogenic and accidental) and tuberculosis.

The causes of hemorrhagic pleural effusion are divided into the following eight groups:

1. Pleuro pulmonary infections (*M. tuberculosis,*[8–10] bacterial pneumonia, e.g. anthrax,[11] brucella,[12] *Klebsiella pneumoniae*[13] and viral, e.g. dengue hemorrhagic fever.
2. Pleuropulmonary malignancy [bronchogenic carcinoma,[14–15] leukemia (acute and chronic),[16–17] pleural tumor (myofibroblastoma,[18] pleural hemangioma[19]), lymphoma, e.g. Hodgkin's lymphoma, non-Hodgkin's lymphoma, angiosarcoma of the chest wall, tumors of the ribs, e.g. osteosarcoma,[20] metastasis to pleura and mediastinal nodes, e.g. breast carcinoma, choriocarcinoma,[21] malignant melanoma,[22] hypernephroma,[23] retroperitoneal chondrosarcoma[24] and bony tumor, e.g. Ewing sarcoma].
3. Connective tissue diseases, e.g. SLE.[25]
4. Asbestos associated, both in benign as well as in malignant mesothelioma.[26]
5. Abdominal diseases [(acute pancreatitis, chronic pancreatitis),[27] ovarian tumors – benign (Meig's syndrome[28]) as well as malignant tumor, mediastinal myelolipoma, uremic pleuritis[29] and rarely, diaphragmatic hernia].
6. Cardiovascular (aneurysm rupture,[30] pulmonary infarction, pulmonary thromboembolism,[31–32] postcoronary artery bypass grafting).
7. Bleeding disorder (overdose of anticoagulant,[33] thrombotic microangiopathies,[34] thalassemia intermediate, liver cirrhosis[35]).
8. Miscellaneous causes (superior venal caval syndrome, Kawasaki disease, chronic renal failure and intralobar sequestration).[36–39]

Sarcoidosis-related pleural effusions occur slightly more commonly in the right lung (45%) than in the left lung (33%),[1] but bilateral and massive effusions have been noted. The reason for the right-sided predominance is unclear and is not related to organ involvement (while in the present case pleural effusion was on the left side).

The mechanism of pleural fluid formation in patients with sarcoidosis is presumably similar to that of other infiltrative diseases. Involvement of the pleura may lead to increased capillary permeability. Superior vena cava obstruction,[39] endobronchial sarcoidosis leading to bronchial stenosis and lobar atelectasis,[40] trapped lung[41–42] and lymphatic disruption with the development of chylothorax have been reported as a cause of sarcoid-related pleural fluid.[39] The reasons for the development of the bloody pleurisy might have been derived from vessels that were involved and compressed by the granulomas. Sarcoid-related pleural effusion has been described as both exudates and transudates[41–43] (pleural effusion in the present case was exudative in nature).

The appearance of pleural fluid among most published case series was serious,[42,44–50] followed by serosanguinous[41,44,46,48,51–53] and less commonly yellow,[44,46] whereas the hemorrhagic effusion was observed very rarely.[5–7] A hemorrhagic pleural effusion is a pleural effusion that looks like it is blood. The RBC count is usually greater than 1,00,000/cu mm[54] (while in the present case it was 2,20,000/cu mm). The typical pleural fluid aspiration finding in sarcoid pleural effusion reveals a paucicellular, lymphocyte predominant, with a pleural/serum protein ratio more consistently in the exudative range (as seen in the present case).

Sarcoid pleural effusions may resolve spontaneously or require corticosteroids for resolution. The time of spontaneous resolution is variable, but most resolve in 1–3 months.[45–48,55] In the present case, corticosteroid therapy resulted in marked improvement of the pleurisy as well as the parenchymal infiltrates.

## Conclusion

In conclusion, sarcoidosis should be included in the differential diagnosis of bloody pleural effusion.

## References

[1] Soskel NT, Sharma OP (2000). Pleural involvement in sarcoidosis. Curr Opin Pulm Med.

[2] Aberg H, Bah M, Waters AW (1966). Sarcoidosis complicated by chylothorax. Minn Med.

[3] Gordonson J, Trachtenberg S, Sargent EN (1973). Superior vena cava obstruction due to sarcoidosis. Chest.

[4] Schaumann MJ (1933). Etude anatomo-patholgique et histologique sur les localizations vicerales de la lymphogranulomatose benigne. Bull Soc Fr Dermatol Syphiligr.

[5] De Vuyst, P, DeTroyer A, Vernault JC (1979). Bloody pleural effusion in a patient with sarcoidosis. Chest.

[6] Takahashi N, Enomoto T, Hagiwara T, Horie T, Amagi S, Tanaka N (1992). A case of sarcoidosis presenting with Heerfordt's syndrome, associated with hepatosplenomegaly, pleural effusion, and ascites. Nihon Kyobu Shikkan Gakkai Zasshi.

[7] Watarai M, Yazawa M, Yamanda K, Yamamoto H, Yamazaki Y (2002). Pulmonary sarcoidosis with associated bloody pleurisy. Intern Med.

[8] Villena V, López-Encuentra A, García-Luján R, Echave-Sustaeta J, Martínez CJ (2004). Clinical implications of appearance of pleural fluid at thoracentesis. Chest.

[9] Seiji M, Kazumi I (2006). A case of hemorrhagic pleurisy suddenly growing after tuberculous pleuritis. J Jp Surg Assoc.

[10] Renert WA (1971). Hemorrhagic pleural effusion: an unusual finding in tuberculous pleurisy. Conn Med.

[11] Mina B, Dym JP, Kuepper F, Tso R, Arrastia C, Kaplounova I (2002). Fatal inhalational anthrax with unknown source of exposure in a 61-year-old woman in New York city. JAMA.

[12] Al-Anazi AR, Aziz S, Fouda MA (2005). Brucellosis: Haemorrhagic pleural effusion a case report. Med Princ Pract.

[13] Lai CP, Wang JH, Chou TW, Tseng WP (1996). Klebsiella pneumoniae induced mycotic aneurysm of the abdominal aorta complicated by bloody pleural effusion: A case report. Jpn Circ J.

[14] Prabhudesai PP, Mahashur AA, Mehta N, Ajay R (1993). Exudative pleural effusions in patients over forty years of age: An analysis of seventy-six patients. J Postgrad Med.

[15] Ong KC, Indumathi V, Poh WT, Ong YY (2000). The diagnostic yield of pleural fluid cytology in malignant pleural Effusions. Singapore Med J.

[16] Tucker DL, Beresford CH, Sigler L, Rogers K (2004). Disseminated beauveria bassiana infection in a patient with acute lymphoblastic leukemia. J Clin Microbiol.

[17] Zeidman A, Yarmolovsky A, Djaldetti M, Mittelman M (1995). Hemorrhagic pleural effusion as a complication of chronic lymphocytic leukemia. Haematologia (Budap).

[18] Kubal C, Ghotkar S, Gosney J, Carr M (2007). A case of Pleural inflammatory myofibroblastoma: A locally aggressive intra-thoracic tumour. J Cardiothorac Surg.

[19] Nanaware S, Gothi D, Joshi JM (2003). Hemorrhagic pleural effusion due to pleural hemangioma. J Assoc Physicians India.

[20] Nakamura A, Yamada Y, Yamamoto T, Yamamoto K, Takeuchi T (1993). A case of osteosarcoma of the rib with bloody pleural effusion. Nihon Kyobu Shikkan Gakkai Zasshi.

[21] Seetharaman ML, Arora R, Arora VK (1991). A case of gestational choriocarcinoma with haemorrhagic pleural effusion is described. Indian J Chest Dis Allied Sci.

[22] Kiser AC, Egan TM (2002). Metastatic melanoma to the pleural space. Ann Thorac Surg.

[23] Gerle R, Felson B (1963). Metastatic Endobronchial Hypernephroma. Chest.

[24] Oyemade OA, Riddick L (1979). Retroperitoneal chondrosarcoma presenting with pleural effusion: A case report. J Natl Med Assoc.

[25] Good JT, King TE, Antony VB, Sahn SA (1983). Lupus pleuritis. Clinical features and pleural fluid fluid antinuclear antibodies characteristics with special reference to pleural. Chest.

[26] Villena Garrido V, López Encuentra A, Echave-Sustaeta J, Alvarez Martínez C, Rey Terrón L, Sotelo MT (2004). Pleural mesothelioma: Experience with 62 cases in 9 years. Arch Bronconeumol.

[27] Namazi MR, Mowla A (2004). Massive right-sided hemorrhagic pleural effusion due to pancreatitis: A case report. BMC Pulm Med.

[28] Agaba EI, Ekwempu CC, Ugoya SO, Echejoh GO (2007). Meigs' syndrome presenting as haemorrhagic pleural effusion. West Afr J Med.

[29] Berger HW, Rammohan G, Neff MS, Buhain WJ (1975). Uremic pleural effusion: A study in 14 patients on chronic dialysis. Ann Intern Med.

[30] Gandelman G, Barzilay N, Krupsky M, Resnitzky P (1994). Left pleural hemorrhagic effusion: A presenting sign of thoracic aortic dissecting aneurysm. Chest.

[31] Bynum LJ, Wilson JE (1976). Characteristics of pleural effusions associated with pulmonary embolism. Arch Intern Med.

[32] Romero Candeira S, Hernández Blasco L, Soler MJ, Muñoz A, Aranda I (2002). Biochemical and cytologic characteristics of pleural effusions secondary to pulmonary embolism. Chest.

[33] Bartziota EV, Naylor B (1986). Megakaryocytes in a hemorrhagic pleural effusion caused by anticoagulant overdose. Acta Cytol.

[34] Patnaik MM, Deshpande AK, Nagar VS, Algotar KM (2004). Thrombotic microangiopathies presenting as an obstetric emergency. J Assoc Physicians India.

[35] Warembourg H, Niquet G, Ducloux G, Basin B (1962). Apropos of 2 cases of hemorrhagic pleural effusion in great abundance in cirrhotics. Lille Med.

[36] Oxman LM (1974). Intralobar sequestration causing hemoptysis and hemothorax. N Y State J Med.

[37] D'Souza S, Khubchandani RP, Shetty AK (2006). Kawasaki disease presenting with hemorrhagic pleural effusion. J Trop Pediatr.

[38] Rice TW, Rodriguez RM, Barnette R, Light RW (2006). Prevalence and characteristics of pleural effusion in superior venal caval syndrome. Respirology.

[39] Gordonson J, Trachtenberg S, Sargent EN (1973). Superior vena cava obstruction due to sarcoidosis. Chest.

[40] Poe RH (1978). Middle-lobe atelectasis due to sarcoidosis with pleural effusion. N Y State J Med.

[41] Heidecker JT, Judson MA (2003). Pleural effusion caused by a trapped lung. South Med J.

[42] Claiborne RA, Kerby GR (1990). Pleural sarcoidosis with massive pleural effusion and lung entrapment. Kans Med.

[43] Schaumann MJ (1933). Etude anatomo-patholgique et histologique sur les localizations vicerales de la lymphogranulomatose benigne. Bull Soc Fr Dermatol Syphiligr.

[44] Chusid EL, Siltzbach LE (1974). Sarcoidosis of the pleura. Ann Intern Med.

[45] Wilen SB, Rabinowitz JG, Ulreich S, Lyons HA (1974). Pleural involvement is sarcoidosis. Am J Med.

[46] Sharma OP, Gordonoson J (1975). Pleural effusion in sarcoidosis: A report of six cases. Thorax.

[47] Selroos O (1966). Exudative pleurisy and sarcoidosis. Br J Dis Chest.

[48] Nicholls AJ, Friend JA, Legge JS (1980). Sarcoid pleural effusion: Three cases and review of the literature. Thorax.

[49] Salazar A, Mana J, Corbella X, Vidaller A (1994). Sarcoid pleural effusion: A report of two cases. Sarcoidosis.

[50] Huggins JT, Doelken P, Sahn SA, King L, Judson MA (2006). Pleural effusion in a series of 181 outpatients with sarcoidosis. Chest.

[51] Beekman JF, Zimmet SM, Chun BK, Miranda AA, Katz S (1976). Spectrum of pleural involvement in sarcoidosis. Arch Intern Med.

[52] Berte SJ, Pfotenhauer MA (1962). Massive pleural effusion in sarcoidosis. Am Rev Respir Dis.

[53] Kovant PJ, Donohoe RF (1965). Sarcoidosis involving the pleura. Ann Intern Med.

[54] Light RW, Macgregor MI, Luchsinger PC, Ball WC (1972). Pleural effusions: The diagnostic separation of transudates and exudates. Ann Intern Med.

[55] Durand DV, Dellinger A, Guerin C, Guerin JC, Levrat R (1984). Pleural sarcoidosis: One case presenting with an eosinophilic effusion. Thorax.

